# Transient receptor potential vanilloid 4 channels contribute to the initiation of water-induced swallowing reflexes

**DOI:** 10.1038/s41598-026-51222-6

**Published:** 2026-04-29

**Authors:** Mohammad Zakir Hossain, Hiroshi Ando, Rita Rani Roy, Junichi Kitagawa

**Affiliations:** 1 Department of Oral Physiology, School of Dentistry, Matsumoto Dental University, 1780 Gobara Hirooka, Shiojiri, Nagano, 399-0781 Japan; 2 Department of Biology, School of Dentistry, Matsumoto Dental University, Shiojiri, Japan

**Keywords:** TRPV4 channels, Water-induced swallowing reflex, Superior laryngeal nerve-afferents, Nodose–petrosal–jugular ganglionic complex, Neuroscience, Physiology

## Abstract

The swallowing reflex is essential for the safe transfer of food and liquids from the oral cavity to the esophagus. Water is a strong stimulus for triggering this reflex, yet the molecular mechanisms underlying water-induced activation remain unclear. The aim of this study was to evaluate whether transient receptor potential vanilloid 4 (TRPV4) channels contribute to the water-evoked swallowing reflex. We applied distilled water (DW) or saline to superior laryngeal nerve (SLN)-innervated swallowing-related regions in anesthetized rats, measured swallowing reflexes and SLN activity, and conducted immunohistochemical analyses of TRPV4 expression. DW elicited more frequent swallowing and greater SLN activation than saline. TRPV4 immunoreactivity was observed in epithelial cells, taste bud–like structures, and nerve fibers within the laryngopharyngeal and laryngeal mucosa. DW application also induced c-Fos expression in the nodose–petrosal–jugular ganglionic complex, with ~ 60% of activated neurons coexpressing TRPV4. Pretreatment with the TRPV4 antagonist RN9893 significantly attenuated DW-evoked swallowing and SLN activity. These findings provide integrated physiological, pharmacological, and immunohistochemical evidence that TRPV4 channels contribute to the initiation of water-evoked swallowing reflexes. Our study provides enhanced understanding of TRPV4-mediated sensory regulation of swallowing and suggests that TRPV4 is a potential therapeutic target for oropharyngeal dysphagia.

**Supplementary Information:**

The online version contains supplementary material available at 10.1038/s41598-026-51222-6.

## Introduction

The swallowing reflex is a critical component of the digestive process, facilitating the passage of food and liquids from the oral cavity to the esophagus^1^. It is a highly coordinated and complex sensorimotor function that is essential for maintaining adequate nutrition and hydration, as well as for protecting the airway during ingestion^1–5^. Swallowing requires precise temporal and spatial integration of sensory inputs and motor outputs involving the oral cavity, pharynx, larynx, and esophagus^1,2^. This reflex can be elicited by various stimuli applied to the mucosal surfaces of the oropharyngeal and laryngeal regions^2,6–9^. Sensory information generated at the mucosal level is conveyed via afferent fibers of cranial nerves to the swallowing central pattern generator (sCPG) in the brainstem, as well as to cortical and subcortical regions involved in swallowing control^1,2,6,10^.

Patients with dysphagia often experience difficulty swallowing thin liquids, because such fluids move rapidly through the oropharynx and require precise and timely coordination of airway closure and bolus propulsion^4,11^. Impairment of this coordination markedly increases the risk of aspiration^4,5,11,12^. Aspiration of liquids can lead to recurrent respiratory infections and aspiration pneumonia, which are major causes of hospitalization and mortality, particularly in older adults and patients with neurological disorders^5,12,13^. Accordingly, elucidating the molecular mechanisms underlying liquid-induced swallowing is of substantial clinical importance, as such knowledge may facilitate the development of targeted therapeutic strategies to reduce aspiration risk and improve patient outcomes^6,14^.

Previous studies have demonstrated that applying water to the pharyngeal and laryngeal regions can elicit swallowing in both animals^15–17^ and humans^18–20^. The laryngopharynx and associated laryngeal regions, which are innervated by the superior laryngeal nerve (SLN)—a branch of the vagus nerve—have been shown to play a key role in initiating the swallowing reflex in response to water stimulation^6,15–17,21–26^. Applying water to these regions increases SLN activity and triggers the swallowing reflex^15–17,21–23,25^. Despite these observations, the molecular mechanisms underlying the water-induced swallowing reflex remain incompletely understood. Recent investigations in mice have shown that laryngeal taste buds^27^ and laryngeal neuroendocrine cells^28^ contribute to water-evoked swallowing through the release of ATP in response to luminal stimulation. The released ATP subsequently activates purinergic receptors on adjacent sensory afferent fibers, thereby initiating neural activity that promotes triggering of the swallowing reflex^27–29^. These findings suggest that ATP-mediated purinergic signaling represents a key downstream pathway linking epithelial stimulation to afferent nerve activation^27–29^. However, although ATP release and purinergic receptor activation appear to constitute critical downstream events, the primary molecular sensor responsible for detecting water stimulation has not yet been identified. In particular, it remains unclear how water-induced physicochemical changes at the mucosal surface are initially transduced into intracellular signals that lead to ATP release. Identification of this upstream sensor is essential for clarifying how water-specific stimuli initiate the signaling cascade that ultimately engages afferent pathways and triggers the swallowing reflex. Because water is markedly hypoosmotic relative to extracellular fluid, its application to the mucosal surface is expected to induce osmotic gradients and transient cell swelling. Ion channels that respond to hypoosmotic or volume-related stimuli therefore represent plausible candidates for this upstream sensing mechanism.

Transient receptor potential vanilloid 4 (TRPV4) channels are non-selective cation channels that function as polymodal receptors that respond to various stimuli, including hypoosmotic conditions^30–34^. In a previous study, we observed that activation of TRPV4 channels in SLN-innervated regions using the chemical agonist GSK1016790A effectively induced the swallowing reflex^35^. Given that water is inherently hypoosmotic, it is plausible that it can activate TRPV4 channels when applied to swallowing-related regions. Supporting this hypothesis, earlier studies suggest that the responsiveness of laryngeal receptors to water is partially mediated by its hypoosmotic properties^36–38^. In addition, water entry into the cell can activate TRPV4 channels, which respond to changes in cell volume^39,40^.

The objective of this study was to determine whether TRPV4 channels contribute to initiation of the water-induced swallowing reflex. We hypothesized that exposure of SLN-innervated laryngopharyngeal and associated laryngeal mucosa to water activates TRPV4 channels, leading to excitation of SLN afferent fibers and subsequent initiation of the swallowing reflex. Furthermore, we postulated that pharmacological inhibition of TRPV4 would attenuate water-evoked SLN activity and reduce the frequency of the swallowing reflex, thereby demonstrating a functional contribution of TRPV4 channels as molecular sensors mediating water-induced swallowing. These hypotheses were tested using integrated physiological, pharmacological, and immunohistochemical approaches in an established rat model of reflex swallowing.

## Methods

### Animals

Experiments were conducted using 69 healthy male Sprague-Dawley rats, weighing approximately 300–450 g. Of these, fourteen rats were used for immunohistochemistry and fifty-five for assessment of the swallowing reflex. Comparable numbers of animals have been used in our previous studies within this line of research^41,42^. Animals were randomly allocated to the respective experimental groups. Sample size was determined a priori by power analysis using SigmaPlot software (version 14.0; Systat Software Inc., San Jose, CA, USA) for the primary outcome analyzed by one-way analysis of variance (ANOVA). Variance estimates were derived from preliminary data obtained under comparable experimental conditions. A minimum biologically meaningful difference of 7 units and a conservative residual standard deviation of 2.5 were assumed (alpha = 0.05; power = 0.80). The analysis indicated that five animals per group were required. All experimental procedures were approved by the Intramural Animal Care and Veterinary Science Committee of Matsumoto Dental University (Ref. No. 394). All experiments were performed in accordance with relevant guidelines and regulations. Every effort was made to minimize animal suffering and reduce the number of animals used. Animals were housed in the Matsumoto Dental University animal facility under controlled conditions (temperature: 22 ± 2 °C; relative humidity: 40 ± 5%; light/dark cycle: 12/12 h). Food and water were available *ad libitum*. All procedures adhered to the ARRIVE (Animal Research: Reporting of In Vivo Experiments) guidelines established by the National Centre for the Replacement, Refinement, and Reduction of Animals in Research.

### Surgical preparation and electromyographic recording of the swallowing reflex

Rats were anesthetized with urethane (1.0–1.5 g/kg, intraperitoneally) and positioned in a supine posture. Adequate anesthetic depth was confirmed by the absence of both the righting reflex and the pedal withdrawal response to a toe pinch. During experimental procedures, animals were continuously monitored to ensure adequate anesthetic depth and physiological stability. A midline incision was made in the ventral neck region to expose the trachea. Respiration was maintained via a custom-made cannula inserted into the trachea and directed toward the lungs. A small ventral portion of the trachea was surgically removed just behind the cricoid cartilage to create a window for solution delivery. To isolate the SLNs as the primary neural input for the swallowing reflex, the pharyngeal (IX-ph) and lingual (IX-li) branches of the glossopharyngeal nerve, as well as the pharyngeal (X-ph) and recurrent laryngeal nerve (RLN) branches of the vagus nerve, were bilaterally transected. The bilateral SLNs were left intact during the main experiments to assess their role in triggering the swallowing reflex. In a separate experimental series, the bilateral SLNs were also transected, along with the aforementioned nerves, to confirm the specific involvement of SLNs in the initiation of the swallowing reflex under the experimental conditions.

Electromyographic (EMG) recordings from the mylohyoid muscle, combined with visual confirmation of laryngeal movements, were used to identify and quantify swallowing reflex events. The mylohyoid muscle, along with other supra- and infrahyoid muscles, is activated during initiation of the swallowing reflex^1^. Recording the activity of one or more of these muscles is a well-established method that has been widely adopted as a standardized method for detecting the swallowing reflex^25,35,43,44^. Previous studies, including those from our group, have reliably identified swallowing reflexes based on high-amplitude EMG activity in the mylohyoid muscle in conjunction with visible laryngeal elevation^25,35,42–45^. To confirm that high-amplitude EMG bursts represented swallowing reflexes rather than coughing or other airway defensive responses, respiratory chest movements and EMG activity from both the mylohyoid and rectus abdominis muscles were simultaneously recorded in a subset of animals. Respiratory chest movements were recorded using a piezoelectric transducer (ADInstruments, Inc., Colorado Springs, CO). EMG signals were recorded using bipolar fine-wire electrodes (stainless steel, urethane-coated; Unique Medical Co., Ltd., Tokyo, Japan). A loudspeaker connected to the EMG system facilitated auditory monitoring of mylohyoid muscle activity during data collection. Signals were amplified and band-pass filtered (150 Hz–3 kHz) using a bioelectric amplifier (MEG-5200; Nihon Kohden, Tokyo, Japan). The filtered signals were digitized at a sampling rate of 10 kHz using a Power 1401 data acquisition system (Cambridge Electronic Design Ltd., Cambridge, UK) and stored on a hard drive for subsequent offline analysis.

### Stimulating solutions

The solutions used for stimulation were 0.9% saline (NaCl; Otsuka Pharmaceutical Co. Ltd., Tokyo, Japan) and DW. Each solution was delivered to the swallowing-related regions innervated by the SLN (laryngopharynx and associated laryngeal regions) in a 50-µl volume using a syringe equipped with a blunted 21-gauge needle. A small ventral portion of the tracheal wall just posterior to the cricoid cartilage was surgically removed to create a window that allowed access to the laryngopharynx and associated laryngeal regions. The solutions were applied rostrally through this window to the regions, which are innervated by SLN, and were not introduced into the tracheal lumen or lower airway. Each solution was administered as a single bolus. A bolus approach allowed rapid, reproducible coverage of the entire target mucosal surface. Continuous infusion was avoided because placement and fixation of a tube within the region would have produced sustained mechanical stimulation, thereby confounding interpretation of the responses. A 50-µl volume was selected because it reliably covered the entire laryngopharyngeal and associated laryngeal mucosal surfaces. All solutions were at room temperature (22–24 °C) when applied.

Following each application, reflex responses were recorded for 20 s. The interval between successive solution deliveries was 2–3 min. During this period, the previously administered solution was aspirated, and the target region was thoroughly rinsed with saline to prevent cross-contamination.

### Immunohistochemistry of superior laryngeal nerve-innervated swallowing-related regions

Deeply anaesthetized rats were subjected to transcardial perfusion with saline followed by 4% paraformaldehyde. The swallowing-related regions were dissected then immersed in 4% paraformaldehyde for 3 h. The specimens were subsequently incubated in 30% sucrose until fully submerged to ensure adequate cryoprotection. The samples were then embedded in Tissue-Tek Optimal Cutting Temperature Compound (Sakura Finetek, Tokyo, Japan) and sectioned in the sagittal plane (10 or50 μm) using a Leica CM1850 cryostat (Leica Biosystems, Nussloch, Germany) before being mounted on glass slides. Thicker Sect.  (50 μm) were prepared to enable clearer visualization of relatively large nerve bundles innervating these regions.

The sections were incubated with 5% normal goat serum in 0.01 M phosphate-buffered saline containing 0.05% Triton X-100 for 1 h to minimize nonspecific binding. Subsequently, the sections were incubated with rabbit monoclonal anti-TRPV4 (1:100; Cat# ab259361; Abcam, Cambridge, UK) at 4 °C overnight, followed by incubation with a fluorescent dye-conjugated secondary antibody (Goat anti-rabbit Alexa Fluor 594; Cat# A-11037; Thermo Fisher Scientific, Waltham, MA) for 1 h at room temperature. The sections were then treated with 4ʹ,6-diamidino-2-phenylindole (DAPI) for 10 min to visualize the cell nuclei. Finally, coverslips were applied using mounting medium (PermaFluor; Thermo Fisher Scientific), and the specimens were examined by fluorescence microscopy (BZ-X700; Keyence Corp., Osaka, Japan). The primary antibody used for TRPV4 detection was the same as that employed in our previous study^35^ and the overall immunohistochemical procedures were similar to those previously described^35^.

### Immunohistochemistry analysis of TRPV4 expression in the nodose–petrosal–jugular ganglionic complex in relation to distilled water application-induced c-Fos expression

The rats were anesthetized with urethane (1.0–1.5 g/kg, intraperitoneally), the trachea was carefully exposed, and a small window was created just behind the cricoid cartilage for DW delivery. The SLN, IX-ph, IX-li, X-ph, and RLN were left intact to avoid nerve transection–induced c-Fos expression. Following surgery, the rats were maintained in a supine position for 4 h to minimize surgery-related c-Fos expression, as reported previously^46,47^.

Four hours after surgery, DW was applied to the laryngopharynx and associated laryngeal regions 15 times (50 µL per application, every 2 min). Each application was maintained for 30 s and then gently aspirated, for a total duration of 30 min. Rats were subsequently kept in the supine position for 1 h, given that c-Fos expression is known to peak approximately 1 h following stimulation^48,49^. One hour after the final DW application, the deeply anesthetized rats were perfused transcardially with saline, followed by 4% paraformaldehyde.

For comparison, a separate cohort underwent identical surgical procedures followed by saline application instead of DW. Saline was applied using the same protocol (15 applications, 50 µL each, every 2 min, 30 s per application). Animals were maintained supine for 1 h after saline application, then perfused as described above.

Each ganglion of the nodose–petrosal–jugular ganglionic complex (NPJc) was identified based on established anatomical criteria^41^. The nodose ganglion (NG) was distinguished by its attachment to the main trunk of the vagus nerve, oriented toward the thorax. The jugular ganglion (JG) was located in the direction of the brainstem, opposite the vagal trunk, whereas the petrosal ganglion (PG) was positioned between the NG and JG. To preserve anatomical orientation during embedding, the right or left NPJc was excised, along with a small segment of the vagus nerve trunk. The excised tissue was fixed in 4% paraformaldehyde, immersed in 30% sucrose until it sank, and then embedded in Tissue-Tek O.C.T. Compound (Sakura Finetek, Tokyo, Japan), maintaining its orientation. The tissue was sectioned into 16-µm-thick slices using a cryostat and mounted onto glass slides, allowing reliable identification of each ganglion after sectioning.

Every third section was processed for fluorescent immunohistochemistry, using a combination of indirect and direct methods^50^ to detect TRPV4 expression in c-Fos–expressing neurons. First, the sections were blocked in 0.01 M phosphate-buffered saline containing 5% normal goat serum and 0.05% Triton X-100 for 1 h to minimize nonspecific binding. They were then incubated overnight at 4 °C with rabbit polyclonal anti-c-Fos antibody (1:1000; Cat# ab190289; Abcam, Cambridge, UK), followed by goat anti-rabbit Alexa Fluor 594 secondary antibody (Cat# A-11037; Thermo Fisher Scientific) for 1 h at room temperature. To prevent nonspecific binding of the prior secondary antibody, the sections were subsequently blocked with normal rabbit serum for 1 h at room temperature^50^, followed by incubation with Alexa Fluor 488–conjugated rabbit monoclonal anti-TRPV4 antibody (1:100; Cat# ab315136; Abcam) for 48 h at 4 °C. Cell nuclei were visualized by counterstaining with DAPI for 10 min. The slides were coverslipped and imaged using a fluorescence microscope (BZ-X700; Keyence Corp.). Immunoreactive cells were quantified using ImageJ software (NIH, Bethesda, MD). In saline-treated rats, the total number of c-Fos–positive neurons and neurons coexpressing c-Fos and TRPV4 were quantified across the entire NPJc. In DW-treated rats, neurons positive for both c-Fos and TRPV4, as well as neurons positive for c-Fos but negative for TRPV4, were quantified within the NG, PG, JG, and across the entire NPJc. In addition, the total number of c-Fos–positive neurons across the entire NPJc was quantified.

The trigeminal ganglion (TG) was used as a positive control for the anti-c-Fos and anti-TRPV4 antibodies (Supplemental Fig. 3A). To induce c-Fos expression in the TG, capsaicin (0.05 mM, 50 µL) was injected into the right vibrissal pad and lower lip in anesthetized rats. One hour later, rats were deeply anesthetized and perfused transcardially with saline, followed by 4% paraformaldehyde. The right TG was excised, fixed, cryoprotected, embedded in Tissue-Tek O.C.T Compound, sectioned at 16 μm using a cryostat, and processed identically to the NPJc sections. As a negative control, sections of the NPJc, laryngopharynx, and associated laryngeal regions were incubated with a universal negative control reagent (Cat# ADI-950-231-0025; Enzo Life Sciences, Inc., Farmingdale, NY) instead of primary antibodies.

### TRPV4 antagonist

A TRPV4 antagonist RN-9893 [(2E)-N-(2,3-dihydro-1,4-benzodioxin-6-yl)-3-[4-(1,1-dimethylethyl)phenyl]-2-propenamide] was obtained from Tocris Bioscience, Bristol, UK. The efficacy of RN-9893 as a selective TRPV4 antagonist has been validated previously^51,52^. RN-9893 was dissolved in a solution containing 3% dimethyl sulfoxide (DMSO; Sigma-Aldrich, St. Louis, MO) and 1% Tween 80 (Sigma-Aldrich), then diluted with saline to the final concentration. The vehicle control consisted of the same DMSO-Tween 80-saline mixture without the antagonist. A 50-µl aliquot of the prepared solution was instilled into the laryngopharyngeal and associated laryngeal regions and left in place for 15 min. The solution was aspirated before applying DW.

### Recording superior laryngeal nerve activity

The sternothyroid muscle was bluntly dissected, and the SLN was carefully freed from the surrounding tissues. Both SLNs were transected distally, near their junction with the vagus nerve. A pair of bipolar silver wire electrodes (0.1 mm diameter) was placed unilaterally beneath the SLN. Liquid silicone was applied to secure the electrodes to the nerve, insulate them from surrounding tissues, and prevent desiccation, thereby enabling stable long-term recordings. SLN activity was amplified and integrated with a time constant of 0.3 s. The signals were digitized using a Power 1401 data acquisition system (Cambridge Electronic Design Ltd., Cambridge, UK) and stored for subsequent analysis.

### Lidocaine

To determine whether sensory fibers in the SLN-innervated swallowing region contribute to the DW–induced swallowing reflex, 2% lidocaine (2-(diethylamino)-N-(2,6-dimethylphenyl)acetamide; Xylocaine®, AstraZeneca Ltd., Osaka, Japan) was applied topically to the target region at a volume of 50-µl, 2 min before DW application.

### Data and statistical analysis

SLN responses were analyzed as the area of the integrated response above baseline using Spike2 software (Cambridge Electronic Design Ltd.). The integrated SLN response was calculated in 2-second bins from the onset of solution infusion for 20 s following stimulation. The stable baseline activity recorded for 2 s prior to stimulation was subtracted from each corresponding 2-second response.

For statistical evaluation, data were first tested for normality and homogeneity of variance to determine whether parametric or non-parametric tests were appropriate. A paired *t*-tests was used to compare the number of swallowing reflexes triggered by saline versus DW. The numbers of c-Fos–positive neurons and c-Fos/TRPV4–coexpressing neurons in the entire NPJc were compared between saline and DW conditions using Welch’s *t*-test. One-way repeated-measures ANOVA, followed by Tukey’s post hoc test, was used to compare the number of swallowing reflexes induced by DW with or without prior application of the TRPV4 antagonist or its vehicle. Nerve responses to different stimulating solutions were compared using one-way repeated-measures ANOVA with Tukey’s post hoc test. In addition, paired *t*-tests were used to compare the number of DW-induced swallowing reflexes before and after lidocaine application or SLN transection.

A critical level of alpha = 0.05 was considered statistically significant. Data are presented as mean ± standard deviation (SD). Statistical analyses were performed using SigmaPlot software (version 14.0; Systat Software Inc.). Graphs were generated using GraphPad Prism software (version 10.4; GraphPad Software, San Diego, CA).

## Results

### Topical application of distilled water facilitated the swallowing reflex

**Fig. 1:**
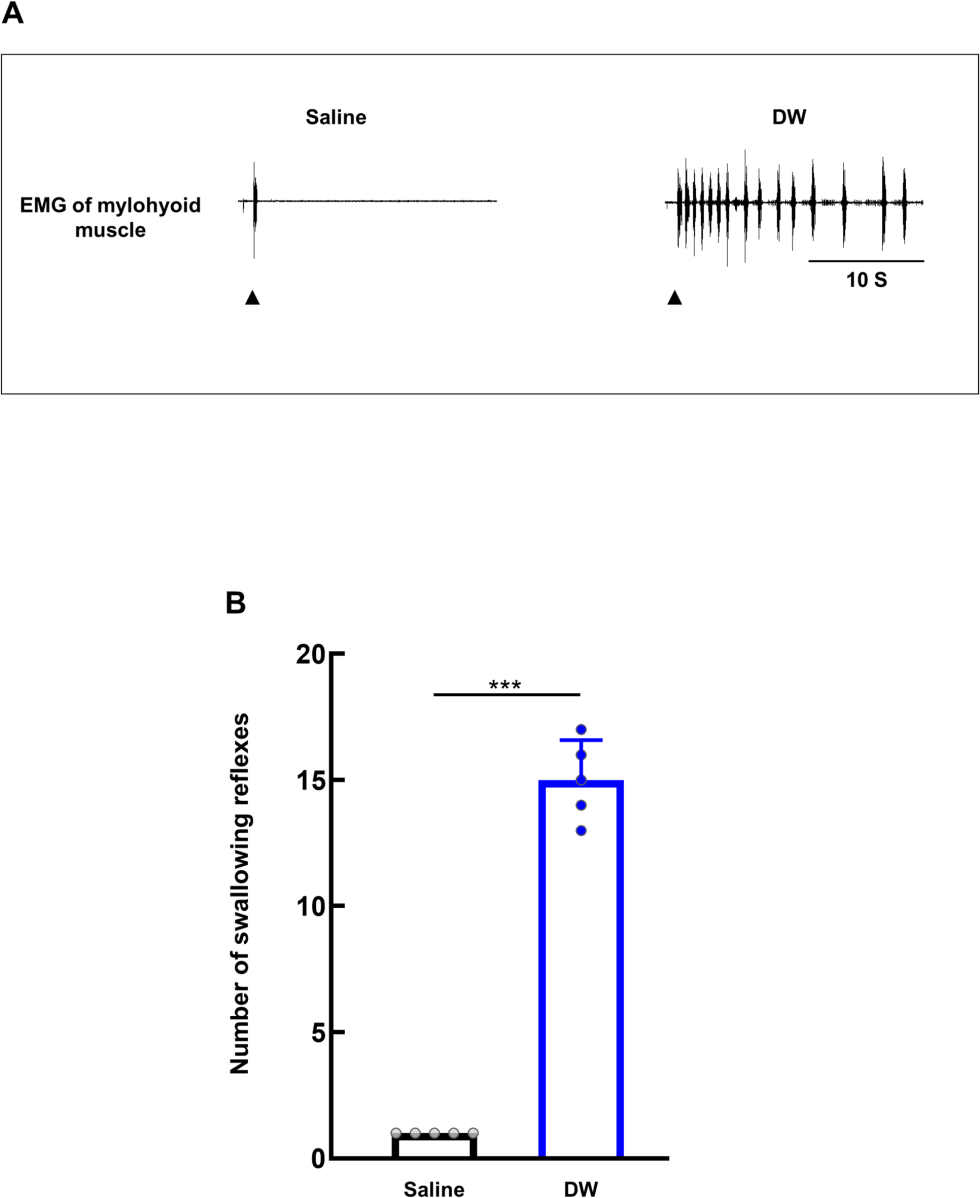
Topical application of distilled water (DW) facilitates swallowing. (**A**) Representative recordings of swallowing reflexes, as detected by electromyographic (EMG) activity of the mylohyoid muscle, following topical application of saline or DW. The black arrowheads indicate when the stimulating solution was delivered. (**B**) Comparison of the number of swallowing reflexes evoked by saline and DW. Statistical analysis was performed using paired t-tests (*n* = 5). Data are presented as the mean ± standard deviation, with individual values shown as points. ***P < 0.001. S, seconds.

We first examined whether DW application facilitated the swallowing reflex. Application of DW to SLN-innervated swallowing-related regions elicited significantly more swallowing reflexes (15 ± 1.58) than saline application (1 ± 0.00), as indicated by high-amplitude EMG bursts in the mylohyoid muscle (paired *t*-test, *t*(4) = 19.80, *P* < 0.001; *n* = 5) (Fig. 1A, B).

To ensure that the observed high-amplitude EMG bursts in the mylohyoid muscle represented swallowing reflexes—rather than coughing or other airway defense responses—respiratory chest movements and EMG activity from both the mylohyoid and rectus abdominis muscles were simultaneously recorded in some animals (Supplementary Fig. 1A, B). The rectus abdominis muscle was included because it is characteristically activated during the cough reflex^53^. Upon DW application to SLN-innervated swallowing-related regions, repetitive high-amplitude EMG bursts were observed in the mylohyoid muscle, accompanied by a temporary cessation of respiration (apnea) (Supplementary Fig. 1B). Notably, no EMG activity was detected in the rectus abdominis muscle. The absence of both respiratory chest movements and rectus abdominis activity confirmed that the EMG bursts in the mylohyoid muscle were due to swallowing reflexes. This is because the cough reflex is typically characterized by inspiratory chest movements followed by forceful expiratory movements accompanying the contractions of abdominal muscles, including the rectus abdominis^53,54^. As respiration gradually resumed, additional swallowing reflexes occurred at increasing intervals, again without rectus abdominis involvement. Saline application induced only a single swallowing reflex, associated with a brief pause in respiration (Supplementary Fig. 1A). These observations confirm that the repetitive EMG bursts observed following DW application specifically represent swallowing reflex activity.

### TRPV4 expression in peripheral swallowing-related regions

**Fig. 2:**
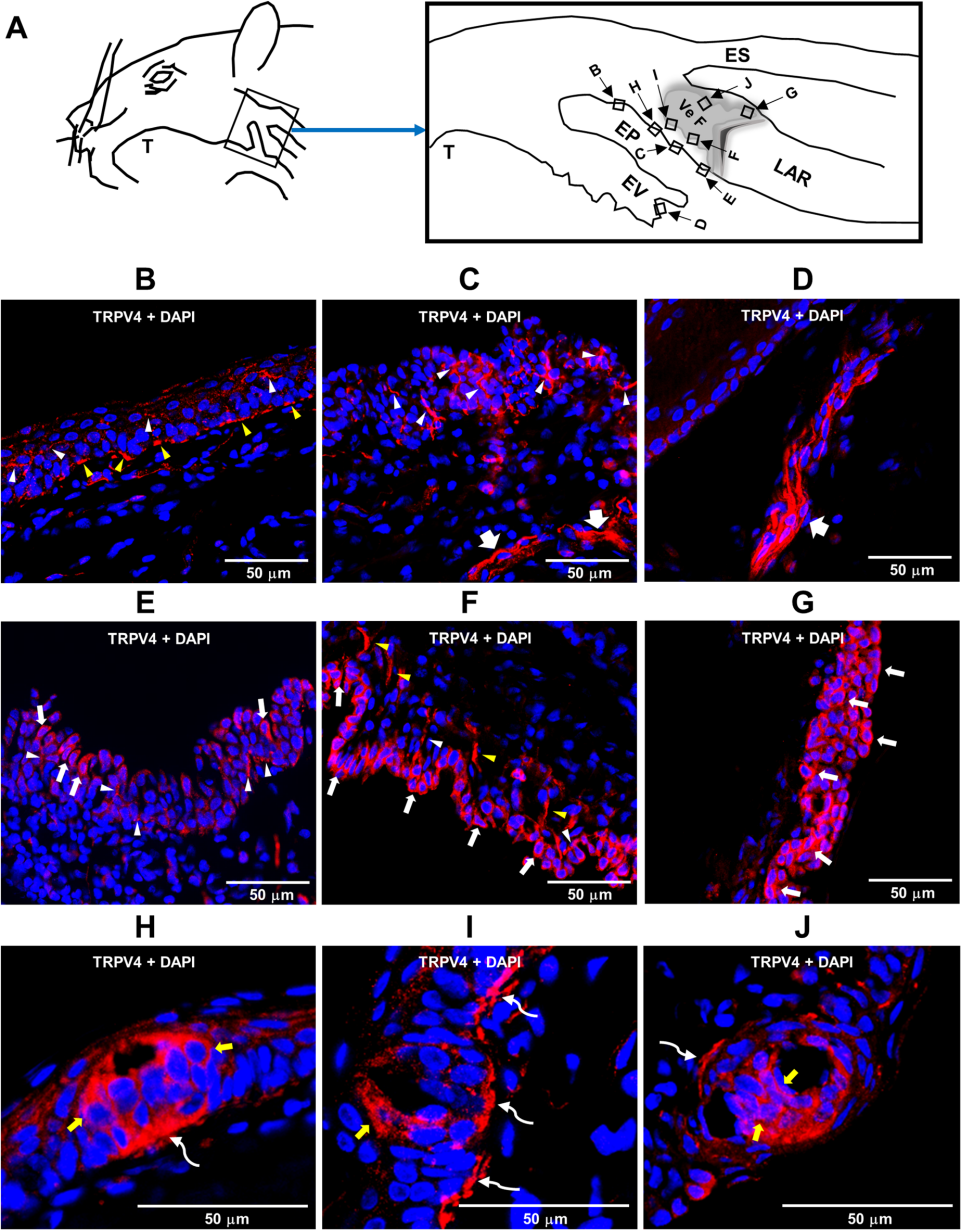
TRPV4 expression in peripheral swallowing-related regions. (**A**) Schematic illustration of the peripheral swallowing-related regions innervated by the superior laryngeal nerve. The boxes in the enlarged image outline the areas from which photomicrographs were obtained. (**B–J**) Representative photomicrographs showing TRPV4 (red) immunoreactivity in the (**B**, **C**) epiglottis (EP), (**D**) epiglottic vallecula (EV), (**E**) base of the epiglottis, (**F**, **G**) vestibular fold (VeF), and (**H–J**) taste bud–like structures. The white arrowheads indicate intraepithelial nerve fibers expressing TRPV4. The thick white arrows denote larger nerves expressing TRPV4. The yellow arrowheads indicate nerve fibers expressing TRPV4 located between the epithelium and lamina propria. The thin white arrows indicate epithelial cells expressing TRPV4. The yellow arrows highlight TRPV4-expressing taste bud cells. The curved white arrows indicate nerve fibers or subgemmal neurogenic plaques associated with taste buds expressing TRPV4. TRPV4 expression in peripheral swallowing-related regions was analyzed in three rats, and the photomicrographs shown are representative images. Panels C and D were obtained from 50-µm sections, whereas the remaining panels were derived from 10-µm sections. Taste buds were identified according to established histomorphological criteria, including their characteristic oval or spindle-shaped profile within the epithelium, the presence of densely packed elongated cells extending from the basal lamina toward the epithelial surface, and their distinct organization relative to surrounding epithelial cells. LAR, larynx; T, tongue; ES, Esophagus.

We next investigated TRPV4 distribution in SLN-innervated swallowing-related regions (Fig. 2) TRPV4 immunoreactivity was observed in both intraepithelial and subepithelial nerve fibers (Fig. 2B–D). In addition, TRPV4 expression was detected in epithelial cells located near the base of the epiglottis, aryepiglottic folds, arytenoids, and vestibular folds (Fig. 2E–G). Furthermore, TRPV4 immunoreactivity was present in the cells of taste bud–like structures, as well as in associated nerve fibers and neurogenic plaques (Fig. 2H–J).

### TRPV4 expression in distilled water application-induced c-Fos–expressing neurons in the nodose–petrosal–jugular ganglionic complex

**Fig. 3:**
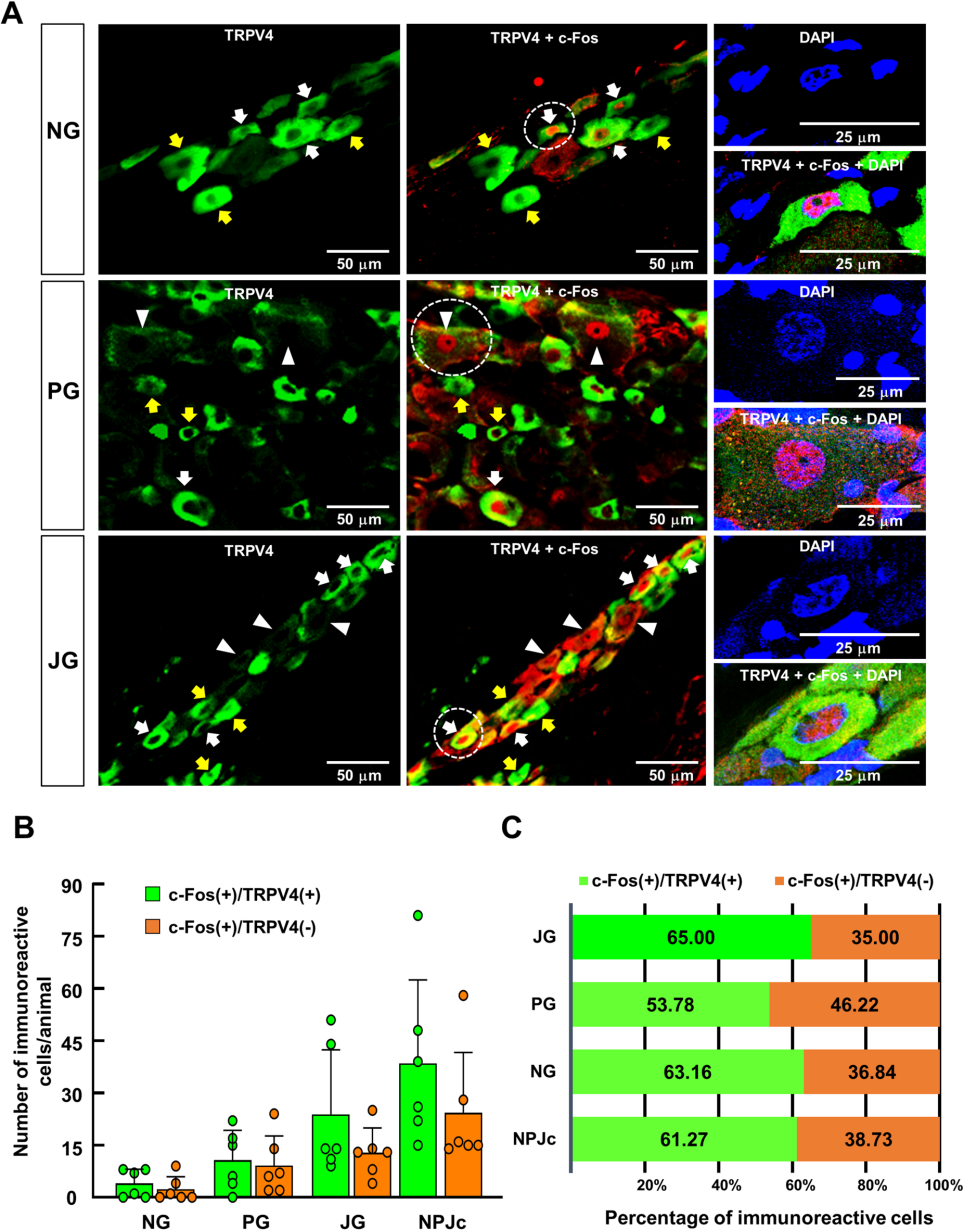
TRPV4 expression in c-Fos–expressing NPJc neurons induced by distilled water application to peripheral swallowing-related regions. (**A**) Representative photomicrographs showing TRPV4 expression in c-Fos–expressing neurons within the nodose ganglion (NG), petrosal ganglion (PG), and jugular ganglion (JG). White arrows indicate neurons that are positive for both TRPV4 and c-Fos, yellow arrows indicate neurons that are positive for TRPV4 but negative for c-Fos, and white arrowheads indicate neurons that are negative for TRPV4 but positive for c-Fos. High-magnification images of the neurons within the dotted circles are shown in the right panels; the nuclei were counterstained with 4ʹ,6-diamidino-2-phenylindole (DAPI). Note that DAPI stained the nuclei of both the neurons and the surrounding satellite cells. (**B**) Quantification of c-Fos–stained, TRPV4-positive, and TRPV4-negative neurons per animal in the NG, PG, and JG and across the entire NPJc. Data are presented as the mean ± standard deviation (*n* = 6). Every third Sect.  (16 μm) of the right or left NPJc from each animal was processed for immunohistochemistry, and the total number of c-Fos–stained TRPV4-positive and TRPV4-negative neurons was counted for each animal. Each dot represents an individual data point. (**C**) Percentage of c-Fos–stained, TRPV4-positive, and TRPV4-negative neurons in the NG, PG, and JG and across the entire NPJc. c-Fos(+)/TRPV4(+), c-Fos–stained neurons immunopositive for TRPV4; c-Fos(+)/TRPV4(−), c-Fos–stained neurons immunonegative for TRPV4.

We then assessed whether TRPV4 was expressed in DW application-induced c-Fos–positive neurons within the NPJc. Following DW application, c-Fos–positive neurons were predominantly observed in the JG (36.67 ± 20.85) and PG (19.83 ± 16.03), with fewer detected in the NG (6.33 ± 7.09) (Fig. 3A, B). Approximately 60% of c-Fos–positive neurons in the NPJc also expressed TRPV4 (Fig. 3C).

It should be noted that the presence of c-Fos–positive neurons in the NPJc cannot be attributed exclusively to DW-induced activation. Mechanical stimulation associated with DW application, subsequent aspiration from the application site, and surgical preparation may also have contributed to neuronal activation, as these factors were unavoidable within the experimental setup of this study.

To determine whether DW induced greater neuronal activation than saline, the numbers of total c-Fos–positive neurons and c-Fos/TRPV4–coexpressing neurons in the entire NPJc were compared between the two conditions (Supplementary Fig. 2). DW application resulted in a significantly greater number of c-Fos–positive neurons compared with saline (Welch’s *t*-test, *t*(5.54) = 3.10, *P* = 0.023; Supplementary Fig. 2B), indicating increased neuronal activation in the NPJc following DW application. Similarly, the number of c-Fos/TRPV4–coexpressing neurons was significantly higher in the DW group than in the saline group (Welch’s *t*-test, *t*(5.64) = 2.82, *P* = 0.032; Supplementary Fig. 2C), indicating enhanced recruitment of TRPV4-expressing neurons following DW application. However, despite this increased expression, it remains technically difficult to isolate neurons activated exclusively by DW under the present experimental conditions.

Because c-Fos is a nuclear marker of neuronal activation expressed^55,56^, only neurons showing nuclear c-Fos expression were included in the counts, although nonspecific cytoplasmic c-Fos staining was also noted. Similar nonspecific cytoplasmic signals were observed on both positive and negative control slides (Supplementary Fig. 3A, B). Therefore, only neurons with nuclear c-Fos expression were included in the counts, even if cytoplasmic expression was also present. Cells with cytoplasmic, but no nuclear, c-Fos expression were excluded. TRPV4 expression was not observed in nuclei.

### Treatment with a TRPV4 antagonist significantly reduced distilled water-induced swallowing reflexes

On the basis of the identification of TRPV4 in peripheral swallowing-related regions and in DW application-induced c-Fos–expressing neurons within the NPJc, we next examined whether a treatment with a TRPV4 antagonist could attenuate DW-induced swallowing reflexes. Various concentrations of the TRPV4 antagonist RN9893 were applied to the swallowing-related regions 15 min prior to DW application. The effects of treatment with a vehicle control (corresponding to the highest concentration of RN9893 used) were also evaluated.

**Fig. 4:**
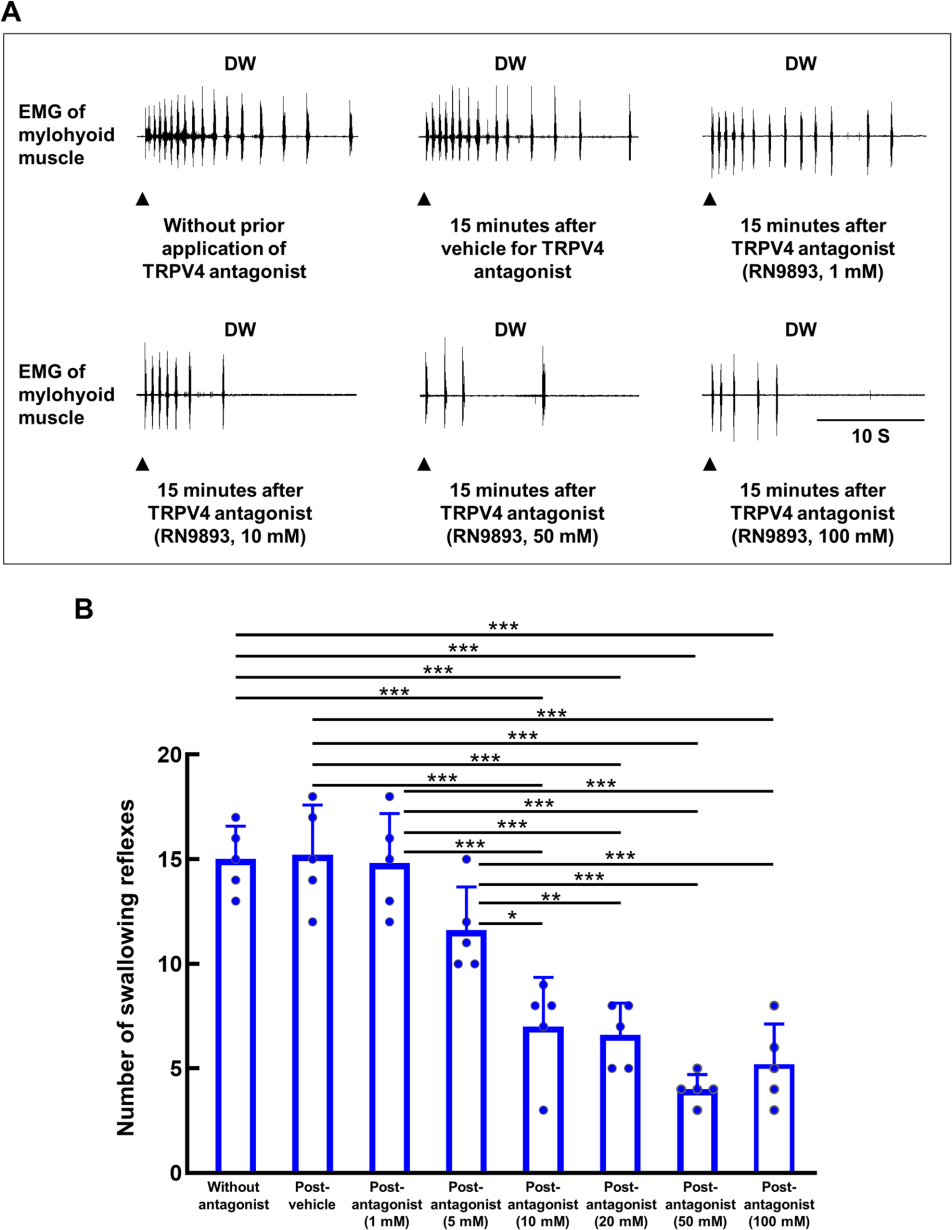
Effect of topical pretreatment with the TRPV4 antagonist RN9893 on the number of distilled water (DW)-induced swallowing reflexes. (**A**) Representative recordings of swallowing reflexes induced by DW following pretreatment with various concentrations of RN9893 or vehicle. Black arrowheads indicate when the stimulating solution was delivered. (**B**) The number of swallowing reflexes induced by DW with and without pretreatment with different concentrations of RN9893 or vehicle. Statistical analysis was performed using one-way repeated measures analysis of variance followed by Tukey’s post hoc test (*n* = 5 for each group). Data are presented as the mean ± standard deviation. *P < 0.05, **P < 0.01, ***P < 0.001. Individual data points are shown as dots. S, seconds; EMG, electromyography.

One-way repeated-measures ANOVA revealed a significant effect of treatment on DW-induced swallowing reflexes (*F*(7,28) = 30.93, *P* < 0.001; *n* = 5). Tukey’s post hoc analysis demonstrated that RN9893 at concentrations of 10, 20, 50, and 100 mM significantly reduced the number of DW-induced swallowing reflexes compared with DW alone (all *P* < 0.001). Similar reductions were observed when these concentrations were compared with the post-vehicle condition (all *P* < 0.001). In contrast, lower concentrations of RN9893 did not produce significant effects. Specifically, the responses at 5 mM did not differ significantly from those observed with DW alone (*P* = 0.130) or after vehicle treatment (*P* = 0.093). Likewise, 1 mM RN9893 produced no significant difference compared with DW alone (*P* = 1.000) or the post-vehicle condition (*P* = 1.000). Comparisons among the higher concentrations (10–100 mM) revealed no significant differences between doses (all *P* ≥ 0.241). Notably, even at the highest concentration tested (100 mM), DW-induced swallowing reflexes were not completely abolished (Fig. 4A, B).

### Treatment with a TRPV4 antagonist significantly reduced distilled water-induced superior laryngeal nerve activity

**Fig. 5:**
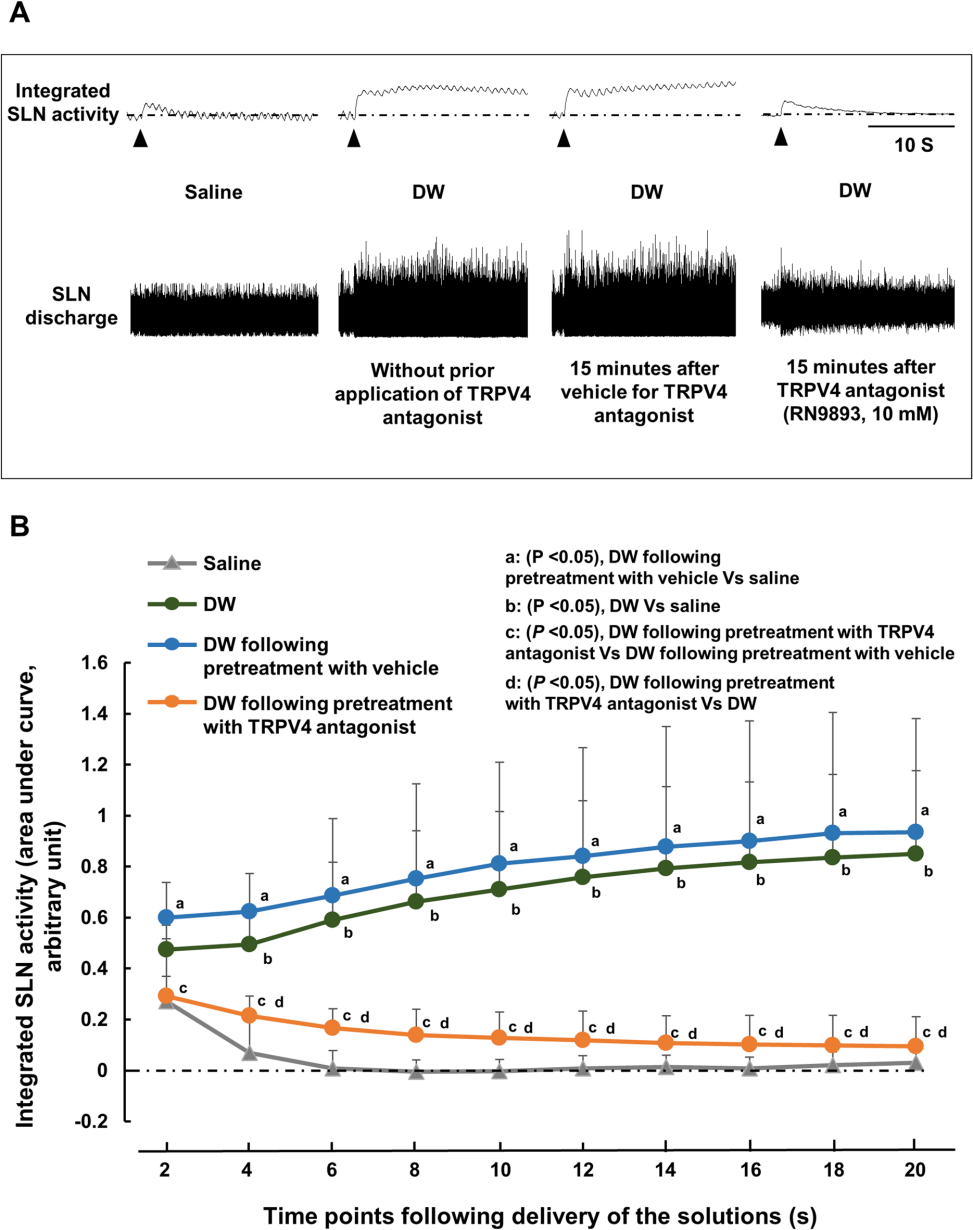
Effect of topical pretreatment with the TRPV4 antagonist RN9893 on distilled water (DW)-induced superior laryngeal nerve (SLN) activity. (**A**) Representative recordings of SLN activity induced by saline, DW alone, DW following pretreatment with vehicle, and DW following pretreatment with the TRPV4 antagonist. Black arrowheads indicate when the stimulating solution was delivered. (**B**) SLN activity induced by saline, DW alone, DW following pretreatment with vehicle, and DW following pretreatment with the TRPV4 antagonist. SLN activity was defined as the area of the integrated response above baseline. The integrated SLN response was calculated in 2-second bins beginning with solution infusion and continuing for 20 s following stimulation. The stable baseline activity recorded for 2 s prior to stimulation was subtracted from each corresponding 2-second response value. Statistical analysis was performed using one-way repeated-measures analysis of variance followed by Tukey’s post hoc test for each 2-second bin among the different groups (*n* = 5). Data are presented as the mean ± standard deviation. S, seconds.

We next asked whether treatment with the TRPV4 antagonist would reduce DW-induced SLN activity. Application of DW markedly increased SLN activity compared with saline application (Fig. 5A, B).

SLN activity was analyzed at 2-second intervals using one-way repeated-measures ANOVA (*n* = 5). A significant treatment effect was detected at 2 s (*F*(3,12) = 4.94, *P* = 0.018). Post hoc analysis revealed that the DW response following vehicle pretreatment was greater than that elicited by saline (*P* = 0.028) and greater than the DW response following RN9893 pretreatment (*P* = 0.040), whereas the response to DW alone did not differ significantly from saline (*P* = 0.225). From 4 to 20 s, significant treatment effects were consistently detected (*F*(3,12) = 15.13–26.44, all *P* < 0.001). During this period, DW significantly increased SLN activity compared with saline (all *P* ≤ 0.002). Pretreatment with RN9893 significantly reduced DW-induced SLN activity compared with both DW alone (all *P* ≤ 0.011) and DW following vehicle pretreatment (all *P* ≤ 0.003). In contrast, vehicle pretreatment did not significantly alter the DW response at any time point (all *P* ≥ 0.301).

### Local anesthetic or bilateral superior laryngeal nerve transection abolished distilled water-induced swallowing reflexes

**Fig. 6:**
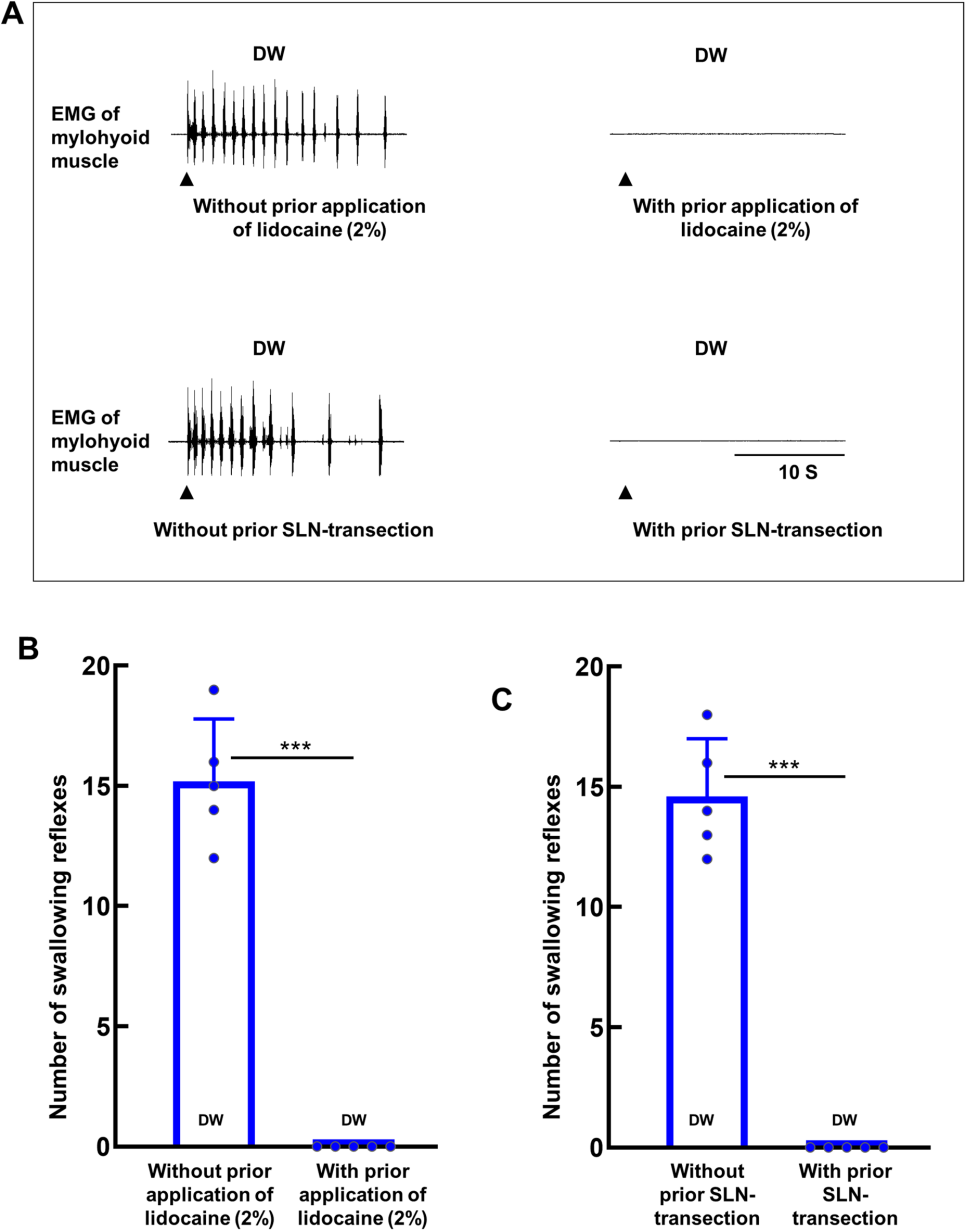
Effect of topical lidocaine administration or bilateral superior laryngeal nerve (SLN) transection on subsequent distilled water (DW)-induced swallowing reflexes. (**A**) Representative recordings of swallowing reflexes triggered by DW with or without prior topical application of 2% lidocaine or bilateral SLN transection. Black arrowheads indicate when DW was delivered. (**B**) The number of swallowing reflexes triggered by DW with and without prior topical lidocaine application. (**C**) The number of swallowing reflexes triggered by DW with and without prior bilateral SLN transection. Statistical analysis was performed using paired *t*-tests (*n* = 5). Data are presented as the mean ± standard deviation, and individual data points are represented by dots. ***P < 0.001. EMG, electromyography.

To confirm that DW-induced swallowing reflexes were mediated by excitation of afferent nerves within swallowing-related regions, a local anesthetic (2% lidocaine) was applied 2 min before DW application. This treatment completely abolished DW-induced swallowing reflexes (paired *t*-test, *t*(4) = 13.13, *P* < 0.001; *n* = 5) (Fig. 6A, B). In addition, no apnea was observed following local anesthetic application (Supplementary Fig. 4A).

Bilateral transection of the SLNs prior to DW application also eliminated the swallowing reflex (paired *t*-test, *t*(4) = 13.56, *P* < 0.001; *n* = 5), demonstrating that SLN afferents mediated the DW-induced response under our experimental conditions (Fig. 6A, C). Similar to the effect of local anesthetic application, no apnea was observed following bilateral SLN transection (Supplementary Fig. 4B).

## Discussion

To the best of our knowledge, this study provides the first evidence that TRPV4 channels are involved in triggering the water-induced swallowing reflex. The enhanced reflex activity elicited by water, compared with saline, supports the hypothesis that water activates specific sensory mechanisms responsible for initiating the swallowing reflex. Our findings collectively demonstrate that TRPV4 channels play a pivotal role in this process.

Immunohistochemical analysis showed that TRPV4 was expressed by epithelial cells, taste bud cells, and nerve fibers within the laryngopharyngeal and laryngeal mucosa—regions that have previously been identified as playing key roles in triggering swallowing. These findings indicate that TRPV4 is strategically positioned to act as a peripheral sensor that responds to mucosal surface changes following water application. Moreover, c-Fos expression induced by water application was observed in the NPJc, with a substantial proportion of activated neurons coexpressing TRPV4. However, mechanical stimulation at the application site—an unavoidable aspect of the experimental procedure—must be considered when interpreting these findings, although measures were taken to minimize such effects, including gentle aspiration and a 4-hour postoperative recovery period in accordance with previous studies. Notably, saline application under identical conditions resulted in significantly fewer c-Fos–positive and c-Fos/TRPV4–coexpressing neurons in the NPJc compared with water. This difference indicates that, although mechanical factors contribute to c-Fos expression, the enhanced activation observed following water application cannot be explained solely by procedural influences. Nevertheless, the contribution of mechanical stimulation to c-Fos expression cannot be completely excluded. In addition, previous studies have reported that mechanically responsive superior laryngeal nerve afferent units are also sensitive to water^22,36,37^, raising the possibility that overlapping populations of neurons are activated by both mechanical and water stimuli. Accordingly, while the present c-Fos analysis demonstrates recruitment of TRPV4-expressing neurons under the distilled water application paradigm, it does not permit definitive separation of water-specific and mechanically induced activation.

TRPV4 localization in peripheral swallowing-related regions is consistent with previous reports describing its expression in nerve fibers of the laryngopharynx and associated laryngeal regions^35^, in epithelial cells of the larynx^57,58^, trachea^59^, and esophagus^60^, and in taste bud cells of the circumvallate papillae^61^. The presence of TRPV4 in these structures suggests its potential role in mediating swallowing-related sensory processes. In our previous study, topical application of the TRPV4 agonist GSK1016790A facilitated the swallowing reflex in a dose-dependent manner^35^. The findings from the present study extend these observations by demonstrating that TRPV4 activation is physiologically relevant for triggering the water-induced swallowing reflex. Pharmacological blockade of TRPV4 with a selective antagonist significantly attenuated both the number of water-induced swallowing reflexes and the associated SLN activity. This attenuation indicates that TRPV4 activation contributes directly to the sensory input driving the water-induced swallowing reflex.

**Fig. 7:**
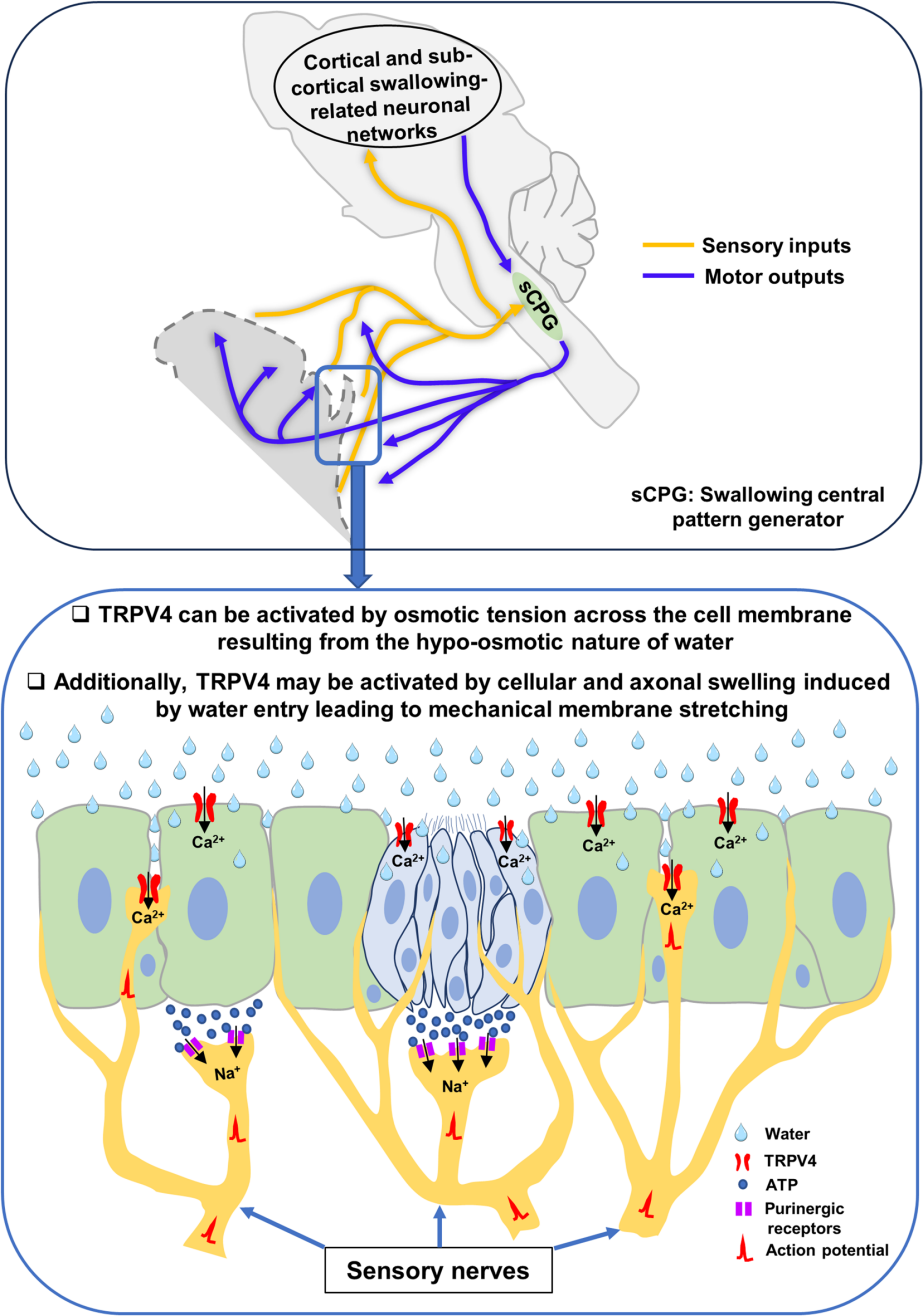
Schematic representation of the proposed mechanisms by which TRPV4 contributes to water-induced swallowing reflexes. Water may generate osmotic tension across the membranes of epithelial cells, taste bud cells, and afferent nerve axons, thereby activating the TRPV4 channels expressed on these structures. In addition, water entry into these cells and axons can induce swelling, leading to mechanical membrane stretching and further TRPV4 activation. TRPV4 activation in epithelial and taste bud cells promotes cation (e.g., Ca²⁺) influx, which triggers ATP release from these cells. The released ATP subsequently activates purinergic receptors on sensory afferent fibers, initiating action potential generation. Furthermore, TRPV4 activation on sensory nerve fibers directly causes cation influx and neuronal excitation. The increased activity of these afferents enhances sensory input to the swallowing central pattern generator (sCPG), ultimately triggering the swallowing reflex.

Several mechanisms may underlie TRPV4 activation following water application (Fig. 7). As a hypo-osmotic sensor^30–32^, TRPV4 can be activated by osmotic tension across the cell membrane resulting from the hypoosmotic nature of water. In addition, TRPV4 may be activated by cellular and axonal swelling caused by water entry through aquaporins or by other mechanisms that induce mechanical stretching of the plasma membrane^39,40^. Previous studies have shown that airway epithelial cells undergo volume changes in response to alterations in luminal osmolarity^62,63^. TRPV4 has been demonstrated to respond to both osmotic and mechanical stimuli^32–34,64,65^. In our SLN recordings, water application produced a gradual increase in neural activity (Fig. 5), suggesting that membrane stretching due to cell or axonal volume expansion may account, at least in part, for the delayed phase of activation. This interpretation is further supported by the greater inhibitory effect of the TRPV4 antagonist on the late component of SLN activity, which is consistent with stretch-induced TRPV4 activation. In agreement with this notion, previous studies have reported that long-latency and long-duration water-responsive SLN afferents did not respond to isotonic solutions, indicating that their activation depends on the hypoosmolarity of water^36–38^.

Previous studies have shown that laryngeal taste buds contribute to water-induced swallowing, likely through ATP release and the consequent activation of purinergic receptors on afferent nerve fibers^27^. However, the specific receptor mediating water-induced activation remained unidentified^29^. Our finding that TRPV4 is expressed in taste bud cells raises the possibility that hypoosmotic stimulation associated with water application may activate TRPV4, leading to intracellular calcium influx and subsequent ATP release. The released ATP could then activate purinergic receptors on afferent fibers, thereby initiating neural signaling. Similarly, a previous study reported that laryngeal neuroendocrine cells—a specialized subset of epithelial cells—are activated by water and release ATP to excite afferent nerves^28^. That study also demonstrated that the neuroendocrine cells are sensitive to hypoosmotic stimulation below 75 mOSm/L^28^. Although neuroendocrine cells were not specifically labeled in the present study, TRPV4 expression in epithelial cells suggests that these populations may also participate in DW-induced signaling.

This model is further supported by our previous finding that P2X3 receptors are predominantly expressed on nerve fibers that innervate the epithelium and taste buds in swallowing-related regions^41^. Other studies have also reported P2X2, P2X3, and P2Y1 receptors expression in these areas^27,29,66–70^. Moreover, water failed to evoke swallowing reflexes in P2X2/P2X3 double knockout mice^27^, reinforcing the importance of purinergic signaling in this process. In addition, application of hypoosmotic solutions has been shown to augment ATP release in animal and human airways^71–74^, while TRPV4 antagonists or gene knockdown impaired hypoosmotic-evoked ATP release from airway epithelial cells^72^. Together with our previous observation that topical application of exogenous ATP elicits swallowing reflexes^41^, these findings support a mechanism in which TRPV4 activation in epithelial and taste bud cells induces ATP release, thereby activating purinergic receptors on afferent fibers (Fig. 7). The ensuing excitation of these afferents enhances sensory input to the swallowing central pattern generator (sCPG) in the brainstem, initiating the swallowing reflex (Fig. 7). Furthermore, TRPV4 expression by intraepithelial and subepithelial nerve fibers suggests that water may also directly activate these fibers (Fig. 7), given that the airway epithelium permits water movement through both transcellular and paracellular pathways^75,76^. Supporting this notion, previous studies have reported that hypoosmotic stimulation depolarized vagal sensory nerves in guinea pigs, mice, and humans—effects that were inhibited by TRPV4 antagonists and attenuated in vagal preparations from *Trpv4−/−* mice^77^.

It should be emphasized that the mechanism illustrated in Fig. 7 represents a hypothesis-driven model based on current evidence and the findings of the present study. Definitive validation of this pathway will require comprehensive investigations, including localization and colocalization analyses of ATP receptors and TRPV4 in the laryngopharyngeal region and NPJc; quantification of ATP release following DW compared with saline application; and systematic evaluation of the effects of ATP release inhibitors and ATP receptor antagonists on DW-induced and TRPV4 agonist–induced swallowing reflexes and SLN activity. Such experiments would represent a substantial and independent line of investigation.

Although treatment with the TRPV4 antagonist significantly reduced the water-induced swallowing reflex in a dose-dependent manner, it did not completely abolish the reflex, even at concentrations of up to 100 mM. This partial inhibition indicates that, in addition to TRPV4, other receptors or ion channels may contribute to the water-induced swallowing reflex. In this context, earlier electrophysiological and behavioral studies have demonstrated that laryngeal and pharyngeal water-sensitive receptors are influenced by the ionic composition of the stimulating solution, particularly by anions such as chloride^18,19,23^. Furthermore, anion channels, including volume-regulated anion channels and calcium-activated chloride channels, have been implicated in epithelial osmosensation and fluid homeostasis, which raises the possibility that such channels may also modulate sensory responses to water^78–80^. Further studies are warranted to elucidate the contributions of these additional mechanisms.

Our findings regarding TRPV4 involvement in the water-induced swallowing reflex have important clinical implications. Patients with oropharyngeal dysphagia often experience difficulty swallowing liquids. Current management strategies primarily rely on compensatory techniques (e.g., modifying bolus viscosity or employing postural adjustments such as the chin-tuck maneuver) and rehabilitative exercises, which have demonstrated limited efficacy^12,81,82^. Consequently, therapeutic strategies that target the sensory modulation of swallowing are urgently required. Recently, chemical neurostimulation, which targets chemosensory ion channels in the peripheral swallowing-related regions including TRP channels, has shown promising results in improving swallowing safety and efficiency in both preclinical and clinical studies^6,14,25,83,84^. Importantly, these findings also highlight the critical role of sensorimotor integration in functional swallowing. Effective swallowing depends on tightly coordinated afferent sensory input and motor output mediated through brainstem and cortical networks^1,2,6,7,10,85–89^. Disruption of this integration is common in populations such as individuals with neurodegenerative diseases, post-stroke dysphagia, and patients with head and neck cancer^89–91^. Impairments in the coordination between sensory input and motor output can exacerbate swallowing difficulties, particularly for thin liquids, underscoring the need for interventions that enhance sensory feedback. Understanding the molecular mechanisms underlying water-induced swallowing reflexes may therefore provide valuable insights that could help develop pharmacological interventions for oropharyngeal dysphagia. Our previous observation that TRPV4 agonists facilitate swallowing^35^ further suggests that these compounds may represent promising therapeutic candidates for patients who experience difficulty in swallowing liquids. Such approaches merit further investigation in clinical contexts.

Topical application of a local anesthetic to the peripheral swallowing-related regions completely abolished the water-induced swallowing reflex, underscoring the essential role of sensory afferents in mediating this response. To delineate the specific contribution of the SLN, we recorded swallowing reflexes in rats with intact SLNs while transecting other nerves, including bilateral IX-ph, X-ph, IX-li, and RLN. In a separate experiment, bilateral transection of the SLNs completely abolished the swallowing reflex elicited by water application to SLN-innervated regions. These findings confirm that SLN afferents play a critical role in transmitting the sensory input needed to initiate the water-induced swallowing reflex under our experimental conditions.

Collectively, the present findings support our hypothesis that exposure to water under the present experimental conditions engages TRPV4 channels in SLN-innervated laryngopharyngeal and associated laryngeal mucosa, leading to excitation of SLN afferent fibers and initiation of the swallowing reflex. The significant attenuation of water-evoked SLN activity and swallowing frequency following pharmacological TRPV4 inhibition provides functional evidence suggesting that TRPV4 contributes to sensory mechanisms underlying water-induced swallowing. While these results substantiate our proposed mechanistic framework, several limitations of the present study should be considered.

Although pharmacological inhibition of TRPV4 significantly reduced the water-induced swallowing reflex and SLN activity, the reflex was not completely abolished, indicating that additional receptors or ion channels, such as anion-sensitive or osmo-/mechanosensitive pathways, may also contribute. Moreover, c-Fos expression in the NPJc was used as an indicator of neuronal activation; however, unavoidable mechanical stimulation during surgical preparation, fluid application, and aspiration may have independently induced c-Fos expression, making it difficult to attribute neuronal activation specifically to water. While this limitation precludes definitive attribution, it does not undermine the interpretation of the findings, as previous studies indicate that overlapping neuronal populations may be activated by both mechanical and water stimuli^22,36,37^. Finally, although TRPV4 was detected in epithelial cells, taste bud cells, and nerve fibers, direct evidence linking TRPV4 activation to ATP release in the laryngopharyngeal and associated laryngeal mucosa is lacking. The proposed model of TRPV4-dependent ATP release and subsequent purinergic receptor activation therefore remains inferential and requires validation through quantitative ATP measurements, receptor colocalization analyses, and pharmacological or genetic manipulation of purinergic signaling pathways. Future studies addressing these points are warranted.

In conclusion, our findings indicate that TRPV4 channels contribute to the initiation of water-evoked swallowing reflexes. By showing that TRPV4 is involved in this response, the present study enhances our understanding of the sensory regulation of swallowing and highlights TRPV4 as a potential therapeutic target for improving swallowing function in patients with oropharyngeal dysphagia.

## Supplementary Information

Below is the link to the electronic supplementary material.

Supplementary Material 1

## Data Availability

The data underlying this article will be made available to other researchers from the authors upon reasonable request.
