# Supplementary material for: Transient receptor potential vanilloid 4 channels contribute to the initiation of water-induced swallowing reflexes
Source: Sci Rep. 2026 Apr 29;16:19967. doi: 10.1038/s41598-026-51222-6 (PMC13319780; doi:10.1038/s41598-026-51222-6)
Supplement: Supplementary file 1 — Supplementary Material 1 [file 41598_2026_51222_MOESM1_ESM.pdf]

Transient receptor potential vanilloid 4 channels contribute to the initiation of water-induced swallowing reflexes

Mohammad Zakir Hossain, Hiroshi Ando, Rita Rani Roy & Junichi Kitagawa

Supplementary figures with figure legends

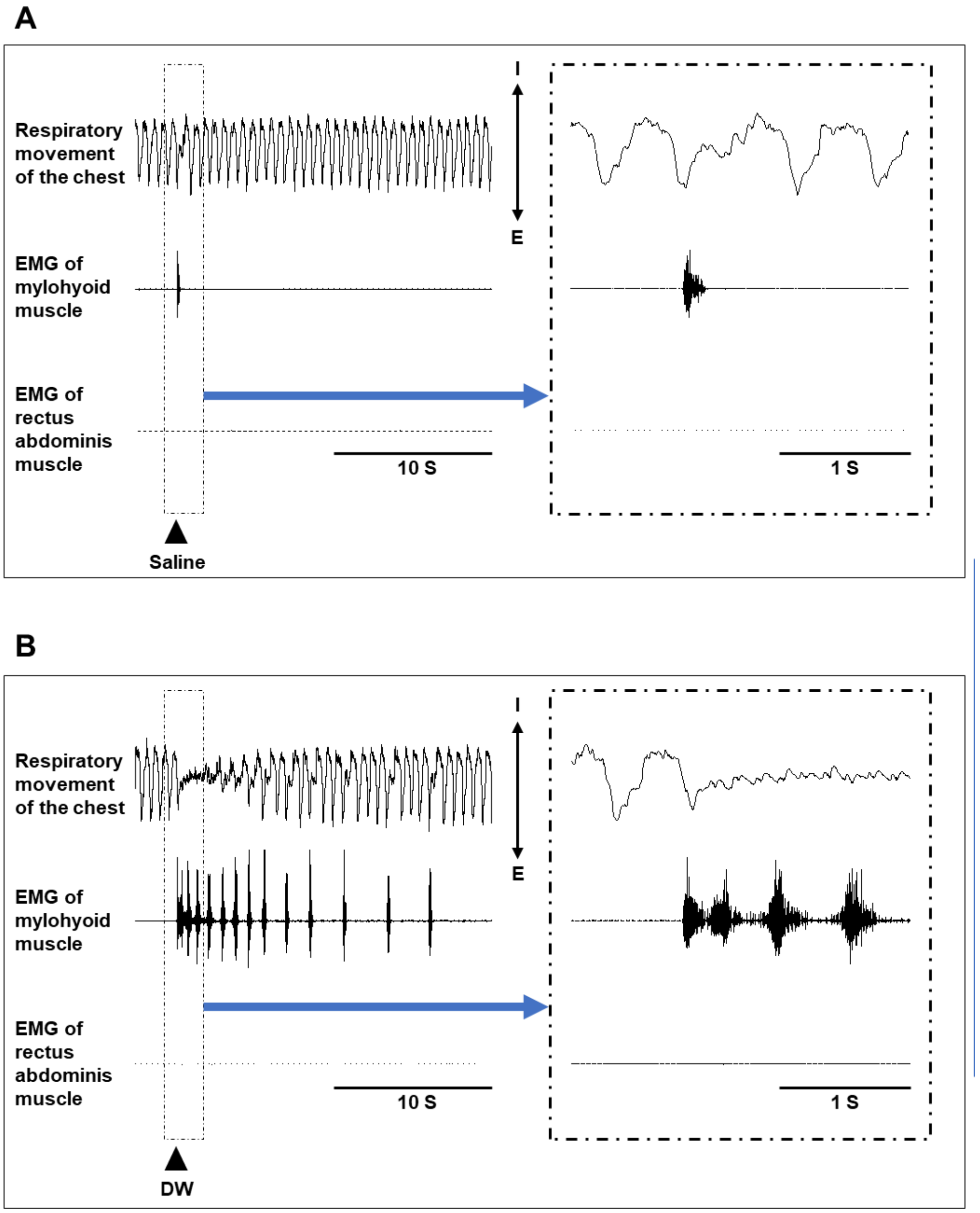

Supplementary Figure 1. Respiratory chest movements and electromyographic (EMG)

activity of the mylohyoid and rectus abdominis muscles following saline and distilled water (DW) administration. **(A)** Representative recordings of respiratory chest movements and EMG activity of the mylohyoid and rectus abdominis muscles after saline administration. The black arrowhead indicates when the saline was delivered. Saline administration evoked a single high-amplitude EMG burst in the mylohyoid muscle, corresponding to the swallowing reflex. This reflex was accompanied by a brief respiratory pause (apnea) without any detectable EMG activity in the rectus abdominis muscle. The inset shows an expanded view of the section outlined by the dotted line. **(B)** Representative recordings following DW administration. The black arrowhead indicates when the DW was delivered. DW application elicited multiple high-amplitude EMG bursts in the mylohyoid muscle within a short period, accompanied by complete cessation of respiratory movements. No EMG activity was observed in the rectus abdominis muscle. The absence of inspiratory and expiratory chest movements, together with the lack of rectus abdominis activity, confirmed that the high-amplitude EMG bursts represented swallowing reflexes rather than cough reflexes. As respiration gradually resumed after repeated reflex triggering, subsequent swallowing reflexes occurred at longer intervals, again without rectus abdominis activation. The inset shows an expanded view of the section outlined by the dotted line. Laryngeal movements associated with the swallowing reflex were also visually observed. Chest movements were recorded using a piezoelectric transducer, which also detected small, heartbeat-associated movements that became evident during respiratory cessation induced by repetitive swallowing reflexes following DW administration. I, inspiratory direction of chest movement; E, expiratory direction of chest movement; S, seconds.

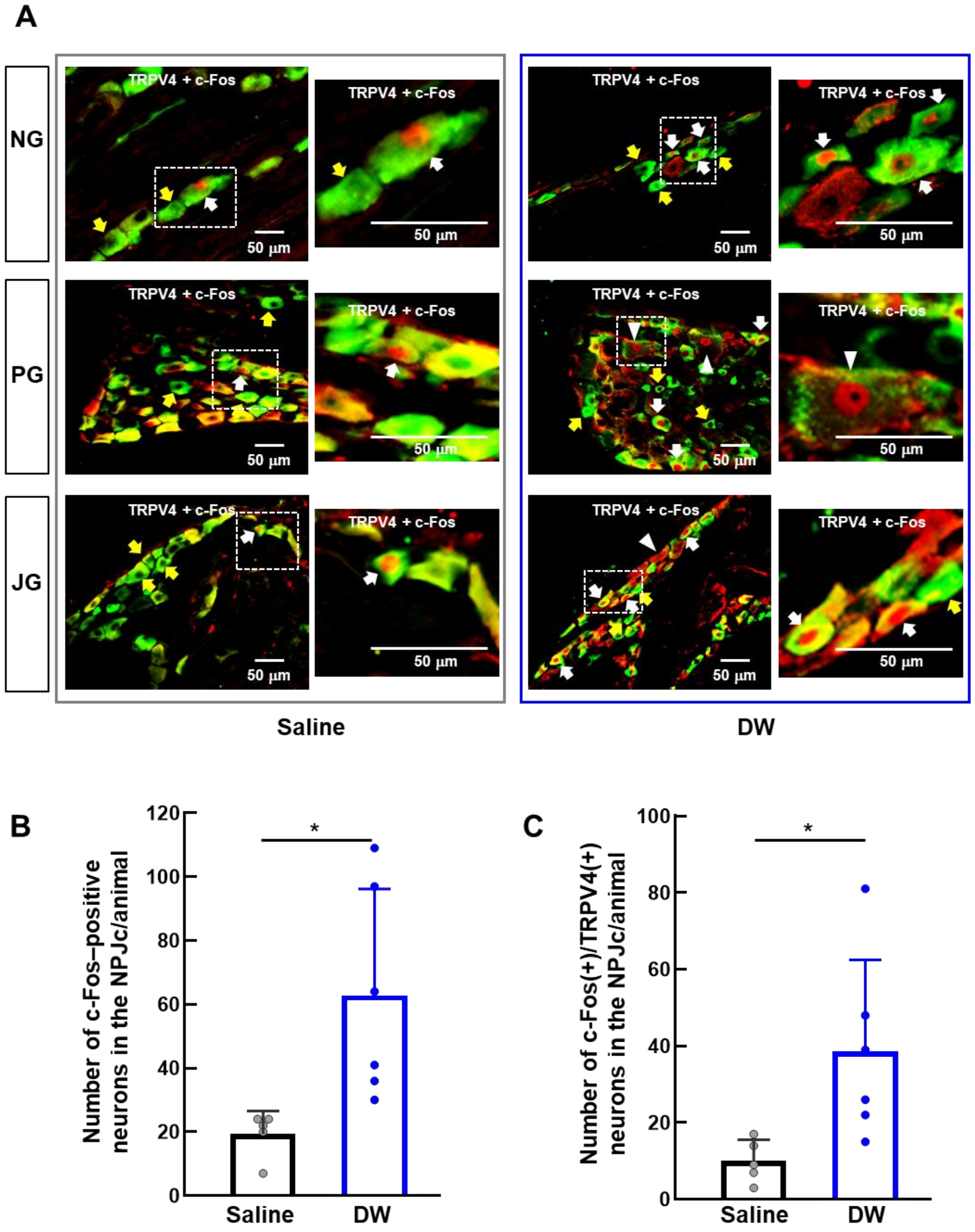

Supplementary Figure 2. Comparison of c-Fos–positive and c-Fos/TRPV4–coexpressing neurons between saline and DW application. **(A)** Representative

photomicrographs showing c-Fos–positive and c-Fos/TRPV4–coexpressing neurons in the nodose ganglion (NG), petrosal ganglion (PG), and jugular ganglion (JG) following saline and DW application. White arrows indicate neurons that are positive for both TRPV4 and c-Fos, yellow arrows indicate neurons that are positive for TRPV4 but negative for c-Fos, and white arrowheads indicate neurons that are negative for TRPV4 but positive for c-Fos. Enlarged views of the regions outlined by dotted lines are shown in the right panels. **(B)** The number of c-Fos–positive neurons in the NPJc was significantly higher following DW application than saline. Statistical analysis was performed using a Welch's *t*-test (n = 5 for saline; n = 6 for DW). Data are presented as mean ± standard deviation (SD). *P < 0.05. Individual data points are shown as dots. **(C)** The number of c-Fos/TRPV4–coexpressing neurons in the NPJc was significantly higher following DW application than saline. Statistical analysis was performed using a Welch's *t*-test (n = 5 for saline: n = 6 for DW). Data are presented as mean ± standard deviation (SD). *P < 0.05. Individual data points are shown as dots.

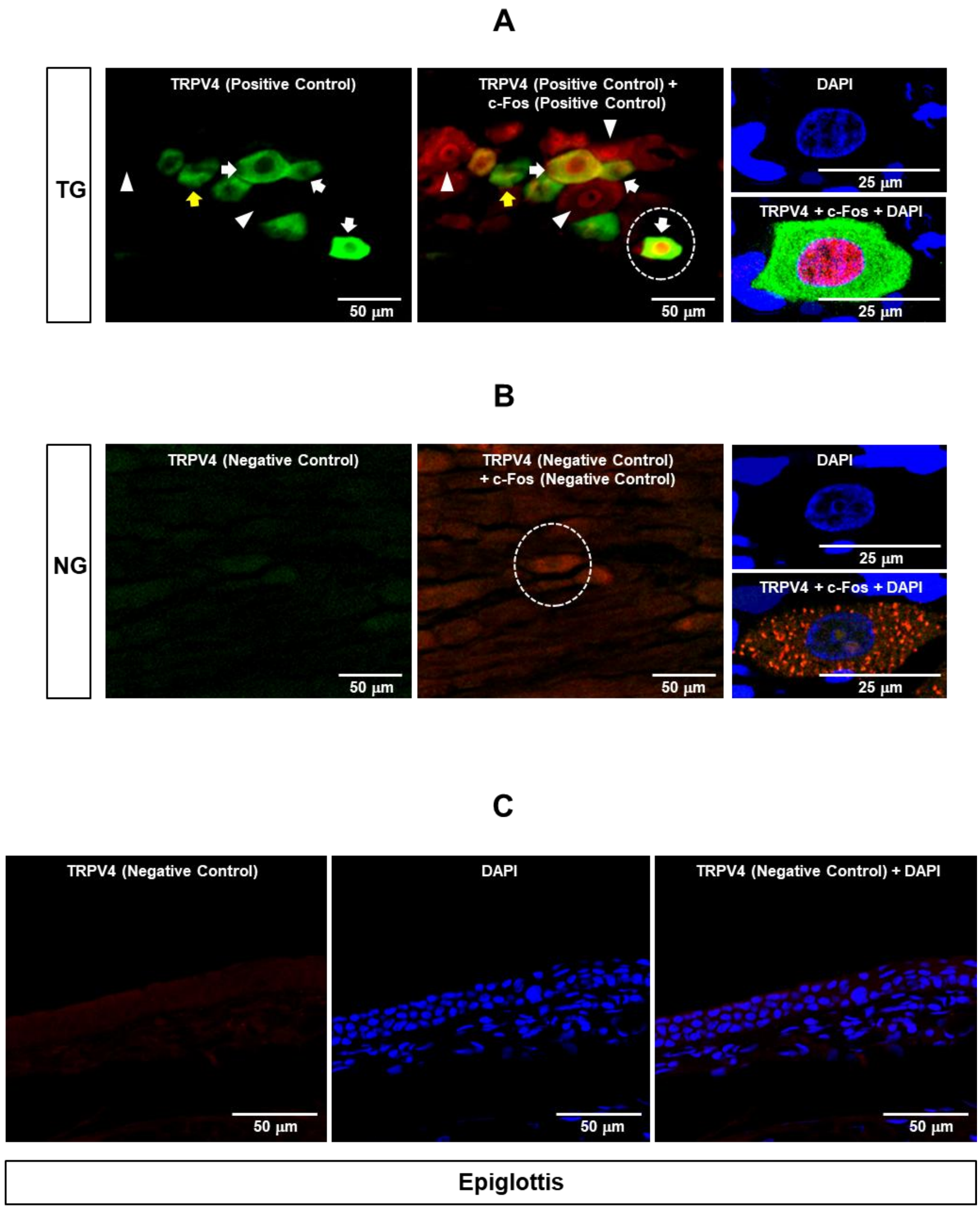

Supplementary Figure 3. Validation of c-Fos and TRPV4 immunostaining using positive and negative controls. **(A)** Representative photomicrograph of a rat trigeminal ganglion (TG) section used as a positive control for the anti-c-Fos (rabbit polyclonal anti-c-Fos

antibody; Cat# ab190289; Abcam, Cambridge, UK) and anti-TRPV4 (Alexa Fluor 488–conjugated rabbit monoclonal anti-TRPV4 antibody; Cat# ab315136; Abcam, Cambridge, UK) antibodies. White arrows indicate cells positive for both c-Fos and TRPV4; yellow arrows indicate neurons positive for TRPV4 but negative for c-Fos; and white arrowheads indicate cells negative for TRPV4 but positive for c-Fos. Nonspecific cytoplasmic c-Fos signals are visible in the positive control section. High-magnification images of the cells outlined by the dotted circles are shown in the right panels; the nuclei were counterstained with 4',6-diamidino-2-phenylindole (DAPI). DAPI stained the nuclei of both the neurons and the surrounding satellite cells. To induce c-Fos expression in the TG, capsaicin (0.05 mM, 50 μL) was injected into the right vibrissal pad and lower lip. **(B)** Representative photomicrograph of a nodose ganglion section that was used as a negative control for the anti-c-Fos and anti-TRPV4 antibodies. A universal negative control reagent containing a mixture of purified rabbit, mouse, and goat immunoglobulins was substituted for the primary antibodies. High-magnification images of the cells outlined by the dotted circles are shown in the right panels, with DAPI nuclear counterstaining. As in the positive control, DAPI stained the nuclei of both the neurons and the surrounding satellite cells. Nonspecific cytoplasmic c-Fos signals were observed in the negative control section. **(C)** Representative photomicrographs of a section from the swallowing-related region (epiglottis) innervated by the superior laryngeal nerve that was used as an additional negative control for the anti-TRPV4 antibody. DAPI staining was used to visualize cell nuclei. As with the nodose ganglion, a universal negative control reagent containing a mixture of purified rabbit, mouse, and goat immunoglobulins was substituted for the primary antibody.

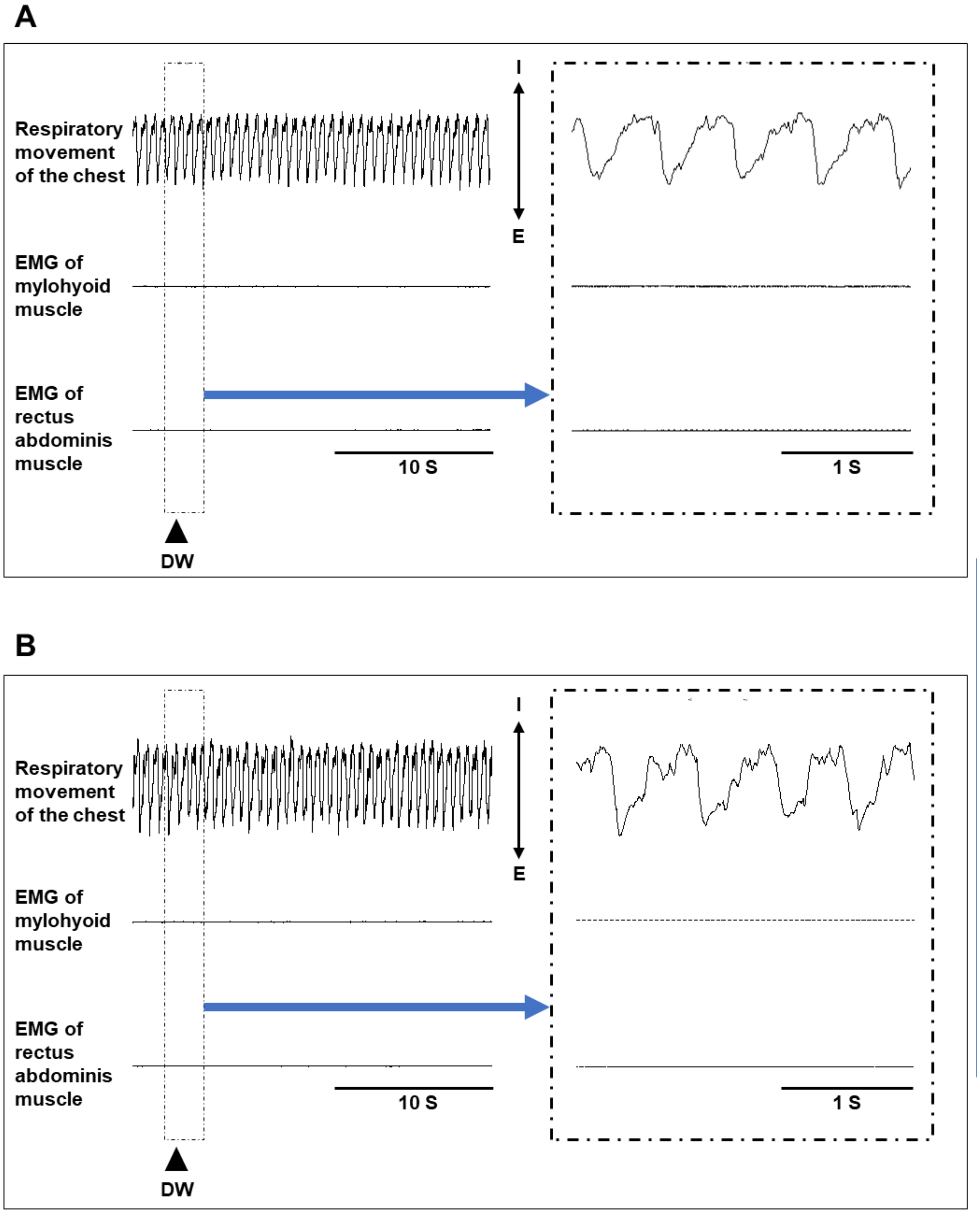

Supplementary Figure 4. Prior topical lidocaine application or bilateral superior laryngeal nerve (SLN) transection prevented distilled water (DW) from inducing swallowing and

apnea. **(A)** Representative recordings of respiratory chest movements and electromyographic (EMG) activity of the mylohyoid and rectus abdominis muscles following DW administration after topical lidocaine application. The black arrowhead indicates when the DW was delivered. No swallowing reflexes or apnea were observed following DW application. The inset shows an expanded view of the section outlined by the dotted line. **(B)** Representative recordings of respiratory chest movements and EMG activity of the mylohyoid and rectus abdominis muscles following DW administration after bilateral transection of the SLNs. The black arrowhead indicates when the DW was delivered. Consistent with panel A, no swallowing reflexes or apnea were observed after DW application. I, inspiratory direction of chest movement; E, expiratory direction of chest movement; S, seconds.
